# Thrombotische Mikroangiopathien: Relevante Neuigkeiten für den Intensivmediziner

**DOI:** 10.1007/s00063-016-0176-6

**Published:** 2016-06-02

**Authors:** M. Gaggl, C. Aigner, G. Sunder-Plassmann, A. Schmidt

**Affiliations:** Klinische Abteilung für Nephrologie und Dialyse, Universitätsklinik für Innere Medizin III, Währinger Gürtel 18–20, 1090 Wien, Österreich

**Keywords:** Hämolytisch-urämisches Syndrom, Hämolyse, Thrombopenie, Komplementproteine, Thrombotisch-thrombozytopenische Purpura, Hemolytic–uremic syndrome, Hemolysis, Thrombocytopenia, Complement proteins, Thrombotic thrombocytopenic purpura

## Abstract

Die thrombotische Mikroangiopathie (TMA) ist klinisch durch eine mechanische Hämolyse, eine geringradig bis stark ausgeprägte Thrombopenie und ein akutes Nierenversagen charakterisiert. Differenzialdiagnostisch kommen das atypische hämolytisch-urämische Syndrom (aHUS), die thrombotisch-thrombozytopenische Purpura (TTP), das Shiga-Toxin-assoziierte HUS (STEC-HUS, früher typisches HUS), und andere seltene Formen der TMA infrage. Ferner kann im Rahmen von diversen Autoimmunerkrankungen, maligner Hypertonie, Malignomen und Infektionen eine TMA als sekundäres Phänomen entstehen. Pathophysiologisch kommt es beim aHUS zu einer überschießenden Aktivierung des alternativen Wegs des Komplementsystems. Essenziell ist daher eine rasche Klärung der zugrunde liegenden Ursache der TMA und eine entsprechende Therapie der Grundkrankheit bei den wesentlich häufigeren sekundären TMA. Bei der TTP ist eine rasche Initiierung von Plasmainfusionen bzw. Plasmaaustausch unumgänglich. Für komplement-mediierte Formen bestehen als etablierte Therapie der Plasmaaustausch und als moderne sehr erfolgreiche Therapieoption Antikomplementtherapien.

## Abstract (en)

Thrombotic microangiopathy (TMA) is a clinical syndrome that is characterized by hemolysis, thrombocytopenia, and acute kidney injury, known as atypical hemolytic syndrome (aHUS), thrombotic thrombocytopenic purpura (TTP), and shigatoxin-associated HUS (STEC-HUS) among others. Several diseases, like malignoma, infections, malignant hypertension, or autoimmune disease can result in secondary TMAs. aHUS is caused by a hyperactivated complement system. Identification of the underlying causes of the TMA is the most important issue and directly associated with treatment success. In case of secondary TMAs, treatment of the actual disease is the most important step, while in case of complement-mediated HUS treatment of choice is plasma exchange or anticomplement agents. For the treatment of TTP, rapid initiation of plasma exchange or plasma infusion is the treatment of choice. Patients with STEC-HUS should solely receive supportive treatment.

Die thrombotische Mikroangiopathie (TMA) ist ein meist akut auftretender Symptomenkomplex am Ende verschiedenster Ätiologien, der sich durch mechanische hämolytische Anämie, Thrombopenie und histopathologisch nachweisbare Mikrothrombosen in den Arteriolen und Kapillaren, v. a. in den Nieren, auszeichnet (Abb. 1; [1, 2]).

**Figure 1:**
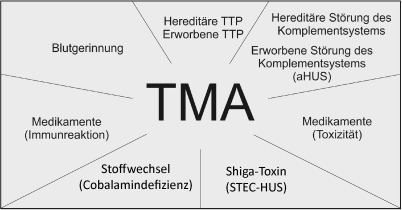

Klinisch präsentiert sich das Vollbild der TMA mit plötzlichem Hämoglobinabfall, Fragmentozyten im Differenzialblutbild, vermindertem oder nicht nachweisbarem Haptoglobin sowie einem Anstieg der Laktatdehydrogenase (LDH). Die Thrombozytopenie kann sehr dezent ausgeprägt sein und führt im Gegensatz zu anderen Differenzialdiagnosen sehr selten zu Blutungskomplikationen.

Bei thrombotischer Mikroangiopathie steht klinisch das akute Nierenversagen im Vordergrund

Das klinisch im Vordergrund stehende meist akut auftretende Nierenversagen macht oft eine Dialyse notwendig und kann zur terminalen Niereninsuffizienz führen. Begleitend treten ausgeprägte Hypertonie und weiteren Organmanifestationen auf. Dazu zählen gastrointestinale (Diarrhö), neurologische und kardiale (Angina pectoris) Beteiligungen. Der oft stark eingeschränkte Allgemeinzustand erfordert in vielen Fällen ein intensivmedizinisches Setting.

Die sowohl erworbenen als auch angeborenen TMA sind durch ihre Ätiologie weiter differenzierbar.

## Thrombotisch-thrombozytopenische Purpura

Der Morbus Moschcowitz (thrombotisch-thrombozytopenische Purpura, TTP; [3, 4]) kommt durch eine ADAMTS13-Defizienz zustande und ist antikörpervermittelt erworben oder durch eine Mutation im ADAMTS13-Gen angeboren. Ob die angeborene Störung bereits im Kindes- oder erst im Erwachsenenalter klinisch manifest wird, hängt vermutlich von der funktionellen Änderung des Proteins durch die Mutation ab. Die erworbene TTP ist eine Autoimmunerkrankung. Die Antikörper gegen ADAMTS13 können direkt nachgewiesen werden. Pathophysiologisch ist in beiden Fällen die Spaltung des von-Willebrand-Faktors gestört, wodurch es zum klinischen Bild einer TMA kommt. Im Gegensatz zu anderen TMA-Syndromen entwickeln sich hier jedoch vorrangig neurologische Symptome und nur in Ausnahmefällen ein akutes Nierenversagen.

## Shiga Toxin-vermitteltes hämolytisch-urämisches Syndrom

Das Shiga-Toxin-vermittelte hämolytisch-urämische Syndrom (STEC-HUS; [5, 6]) entsteht durch Escherichia-coli-Bakterien, die Shiga-Toxin produzieren und am häufigsten über O157:H7-kontaminierte Lebensmitteln oder Wasser aufgenommen werden. Meistens erkranken Kinder an Diarrhö, gefolgt von akutem Nierenversagen mit Thrombopenie und hämolytischer Anämie.

Das Shiga-Toxin gelangt über Globotriaosylceramid (Gb3) in Endothelzellen, Pododzyten und Tubuluszellen, inaktiviert dort die Ribosomen und führt zur Apoptose der Zelle. Ferner entsteht durch eine toxinmediierte Sekretion des von-Willebrand-Faktors ein proinflammatorischer und prothrombotischer Zustand. Der Nachweis von Shiga-Toxin im Stuhl ist ein Hinweis auf die Ursache der TMA.

## Komplementvermittelte thrombotische Mikroangiopathie

**Figure 2:**
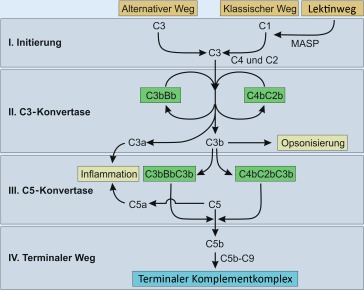

**Figure 3:**
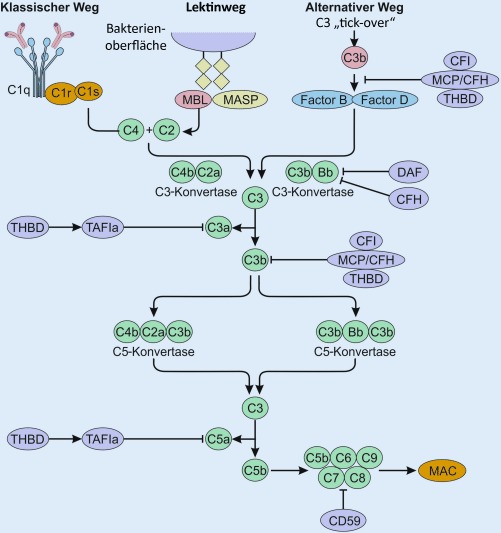

Die komplementvermittelte TMA ([1]; atypisches [a]HUS) entsteht durch eine Dysregulation des alternativen Wegs des Komplementsystems. Diese läuft vereinfacht dargestellt in 4 Schritten ab:

- Initiierung,
- Bildung der C3-Konvertase,
- Bildung der C5-Konvertase und
- Bildung des terminalen Komplementkomplexes

(Abb. 2). Speziell dieser Teil des unspezifischen Immunsystems beinhaltet eine kontinuierliche Aktivierung, die aktiv reguliert werden muss. Bei der TMA kommt es zu einer physiologischen Aktivierung oder Überaktivierung durch Infektionen oder andere Auslöser und in Folge zu einer Inkompetenz der Regulierung und Deaktivierung. C3 wird zu C3a und C3b hydrolysiert. Letzteres bindet an die Zellmembranen. Gemeinsam mit Faktor B wird die C3-Konvertase (C3bBb) gebildet, die sich mit einem weiteren C3 zu C5 verbindet. In weitere Folge entstehen C5a und C5b bzw. C5b-9, der terminale Komplementkomplex („membrane attack complex“; MAC). Die C3-Konvertase wird streng durch Faktor H und Faktor I reguliert und führt so zu einer physiologischen Inaktivierung der Kaskade. Eine membranständige Regulation erfolgt über „membrane cofactor protein“ (MCP), „decay-accelerating factor“ (DAF), Thrombomodulin (THBD) und CD59 (Abb. 3).

Bei den angeborenen Formen besteht am häufigsten eine funktionell wirksame Mutation im Komplementfaktor-H(*CFH*)-Gen. Weitere ursächlich mutierte Gene oder Kombinationen aus solchen sind Komplementfaktor I (*CFI*) und Membrankofaktorprotein (*MCP; CD46*) sowie Mutationen in *C3* selbst oder in dessen Kofaktor Komplementfaktor B (*CFB*). Die Mehrheit dieser genetischen Varianten tritt heterozygot auf und kann oft bei asymptomatischen Familienmitgliedern nachgewiesen werden. Die Penetranz der Erkrankung liegt bei Mutationsträgern nur bei 50 %. Daher sind die genetischen Befunde äußerst vorsichtig zu interpretieren und Familienscreenings nur in ausgewählten Fällen empfehlenswert.

Bei den erworbenen Formen der komplementvermittelten TMA kommt es meistens zu Antikörperbildung gegen CFH. Dies führt zu einer funktionellen Defizienz des regulatorischen Proteins, das keine suffiziente Deaktivierung der C3-Konvertase bewirken kann.

## Sekundäre thrombotische Mikroangiopathien

Mögliche Primärerkrankungen oder Trigger einer sekundären TMA [2] sind Autoimmunerkrankungen (systemischer Lupus erythematodes, Antiphospholipidsyndrom, systemische Sklerose), Schwangerschaft (Präeklampsie, Eklampsie, HELLP-Syndrom: „hemolysis, elevated liver enzyme levels, low platelet levels“), Infektionen (Zytomegalie, Influenza-A-Virus H1N1, HIV, Aspergillus, Pneumokokken), hypertensive Entgleisungen und Malignome. Ferner kann eine TMA nach Stammzell- oder solider Organtransplantationen auslöst werden (Tab. 1).

**Table 1:** 

Kategorie	Mögliche Auslöser
Autoimmun	Systemischer Lupus erythematodes Antiphospholipidsyndrom Sklerodermie Glomerulonephritiden
Infektionen	HIV Influenza-A-Virus H1N1 Sepsis/systemisches inflammatorisches Responsesyndrom
Medikamente	Quinine Mitomycin Gemcitabin Cisplatin Interferon Inhibitoren des „vascular endothelial growth factor“ (VEGF) Tyrosinkinaseinhibitoren Ticlopidin und Clopidogrel Cyclosporin und Tacrolimus Sirolimus Valaciclovir Orale Kontrazeptiva
Andere Erkrankungen	Maligne Hypertonie Malignome Methylmalonacidurie mit Homozysteinurie
Andere Therapien	Bestrahlung Transplantation (Organ und Knochenmark)
Schwangerschaftsassoziiert	HELLP („hemolysis, elevated liver enzyme levels, low platelet levels“), (Prä)eklampsie

Klinisch sind die diversen Formen der sekundären TMA nicht von den primären TMA abzugrenzen, da die gleichen Symptome (akutes Nierenversagen, Thrombopenie, arterielle Hypertension, mechanische Hämolyse) auftreten. Besteht in der Anamnese bereits eine der genannten Grunderkrankungen, ist eine erste Zuordnung der Erkrankung möglich.

## Störungen des Gerinnungssystems

Relativ neu ist die Erkenntnis, dass auch genetische Veränderungen in Thrombomodulin, Plasminogen und Diacylglycerolkinase Ε (DGKE) zu klinischen Bildern der TMA führen können [7–9]. Loss-of-function-Mutationen im DGKE*-*Gen führen zu einer Aktivierung der Proteinkinase C. Diese aktiviert prothrombotische Faktoren (von-Willebrand-Faktor, „tissue faktor“, Plasminogenaktivator-1-Inhibitor).

## Cobalamin-C-Defizienz

Eine Cobalamin-C(Vitamin-B_12_)-Defizienz tritt primär im frühen Kindesalter auf, wurde aber auch schon bei Erwachsenen beschrieben. Sie führt zu einer stoffwechselvermittelten TMA [10]. Ursache dieser Erkrankung sind homozygote oder compound-heterozygote Mutationen im Gen für das Methylmalonic-aciduria-and-homocysteinuria-type-C-Protein (*MMACHC*). Messbar sind ein erhöhter Plasmahomozysteinspiegel und ein verminderter Methioninspiegel. Diese führen zur Aktivierung der Thrombozyten und des Gerinnungssystems und zur endothelialen Dysfunktion.

## Medikamentös-toxische Auslöser

Es gibt die immunologisch vermittelte medikamentös-toxische TMA und die dosisabhängige medikamentös-toxische TMA. [4, 11–14]. Bei einer Therapie mit Quinin, Gemcitabin oder Quetiapin können sich Antikörper bilden, die mit einer Reihe von körpereigenen Strukturen kreuzreagieren. Im Gegensatz dazu kommt es bei einer Therapie mit beispielsweise Kalzineurininhibitoren zu einer dosisabhängigen Endothelaktivierung und so zum klinischen Bild einer TMA. Bei einer Therapie mit Vascular-endothelial-growth-factor(VEGF)-Inhibitoren führt der gewünschte Wirkungsmechanismus zu einer unerwünschten Inhibition von VEGEF an den Podozyten und am renalen Endothel. Das führt zu einer glomerulären TMA mit ausgeprägter Proteinurie.

## Fallbeispiel

Eine 27-jährige Patientin wurde 6 Tage nach der Geburt ihres 2. Kinds in der Notfallaufnahme eines peripheren Krankenhauses vorstellig. Die Geburt war problemlos verlaufen und die Patientin konnte am ersten Tag post partum entlassen werden. Nach rezidivierenden Synkopen während der folgenden Tage wurde sie schließlich wieder stationär aufgenommen. Im Aufnahmelabor fielen eine ausgeprägte Anämie (Hämoglobin: 5,3 mg/dl), eine Thrombopenie (Thrombozyten: 14 G/l) und ein akutes Nierenversagen (Kreatinin: 8,1 mg/dl) auf. Die LDH war mit 2913 I.E./l stark erhöht. Differenzialdiagnostisch wurde bereits an eine TMA gedacht und das C3c im Serum bestimmt (0,67 g/l; Normalwerte 0,9–1,8 g/l), jedoch keine Plasmapheresen durchgeführt.

Aufgrund der zunehmenden Verschlechterung des neurologischen Zustands der Patientin wurde diese schließlich intubiert und auf eine Intensivstation verlegt, wo schließlich die Plasmapherese initiiert wurde. Es stellte sich eine rasche Besserung des Allgemeinzustands der Patientin ein, die jedoch weiterhin hämodialysepflichtig war. Bei ADAMTS13-Werten im Normalbereich wurde eine thrombotisch-thrombozytopenische Purpura ausgeschlossen. Die Thrombozyten und die LDH stabilisierten sich nur langsam nach täglicher Plasmaseperation und hochdosierter Gabe von Kortikosteroiden.

In der Nierenbiopsie zeigte sich eine thrombotische Mikro- und Makroangiopathie

Eine nach Stabilisierung der Gerinnungssituation durchgeführte Nierenbiopsie zeigte eine thrombotische Mikro- und Makroangiopathie mit vorwiegend chronischem Läsionsmuster sowie einen schweren akuten Tubulusschaden und eine bereits mittelgradige interstitielle Fibrose. Nach diesem Biopsieergebnis wurden die Plasmapheresen nach der 29. beendet, da kein Regenerationspotenzial der Niere mehr gesehen wurde.

Nach der Biopsie entwickelte die Patientin eine arteriovenöse (AV-)Fistel, die nach Transfusion von 19 Erythrozytenkonzentraten interventionell versorgt werden musste. Das Zustandsbild der Patientin stabilisierte sich langsam, jedoch wurde sie in den darauffolgenden Tagen respiratorisch instabil und musste schließlich wieder auf die Intensivstation transferiert werden. Differenzialdiagnostisch kamen ein „acute respiratory distress syndrome“ (ARDS), eine transfusionsassoziierte Lungeninsuffizienz (TRALI), aber auch eine Lungenbeteiligung im Rahmen der systemischen thrombotischen Mikroangiopathie infrage. Die Patientin zeigte außerdem eine ausgeprägte schwer zu behandelnde Hypertonie, die schließlich nur mit parenteralen Antihypertensiva einzustellen war, sowie ischämietypische Veränderungen im Elektrokardiogramm (EKG) ohne Korrelat im Serum.

Insgesamt stabilisierte sich das Zustandsbild der Patientin, allerdings blieb sie dialysepflichtig. Nach mehreren durch Insulte und schwere hypertensive Entgleisungen bedingte Intensivaufenthalten konnte die Patientin stabilisiert und nach 2 Jahren erfolgreich nierentransplantiert werden. Nach der Transplantation kam es erneut zu schweren TMA-Episoden, weshalb eine Therapie mit Eculizumab mit gutem Erfolg initiiert wurde.

Die genetische Analyse der Komplementfaktoren wurde erst nach der Transplantation durchgeführt und zeigte eine heterozygote Deletion im Komplementfaktor H, die zu einem Stoppcodon führt, sowie eine krankheitsassoziierte heterozygote Mutation in *MCP* und eine homozygote Mutation im ADAMTS13-Gen.

## Diagnostik

**Figure 4:**
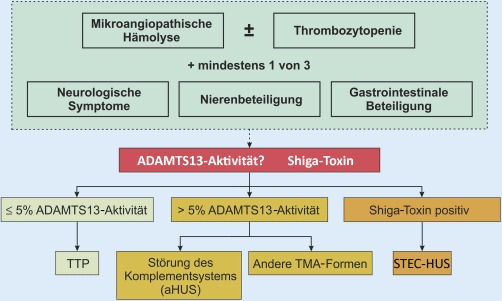

Aufgrund des meistens akuten und dramatischen Krankheitsbeginns und der Unmöglichkeit einer prompten exakten Diagnose ist es essenziell, sich an ein striktes diagnostisches Schema zu halten (Abb. 4). Die Diagnostik ist vor einer etwaigen Therapieeinleitung durchzuführen, da beispielsweise ein Plasmaaustausch wichtige Parameter verfälscht. Primär können durch die Bestimmung der ADAMTS13-Aktivität und von Antikörpern sowie durch den direkten Nachweis des Shiga-Toxins eine TTP oder ein STEC-HUS ausgeschlossen bzw. bestätigt werden.

Ein striktes diagnostisches Schema sollte befolgt werden

Sollte keine der beiden Krankheiten nachgewiesen werden, müssen die anderen TMA-Formen differenzialdiagnostisch bedacht werden: Falls nicht schon anamnestisch bekannt müssen Autoimmun-, Tumor- und Infektionserkrankungen ausgeschlossen werden. Trotz des seltenen Auftretens der stoffwechselvermittelten TMA müssen Vitamin B_12_, Homozystein und Folsäure bestimmt werden. Eine Analyse des Komplementsystems (C3, C4, CH50, falls verfügbar Faktor-H-Antigen, Faktor-I-Antigen, Faktor-B-Antigen, C1q-Autoantikörper) sowie eine Genanalyse der entsprechenden Gene (*CFH, CFI, MCP, C3*, falls verfügbar *CFB, THBD, ADMTS13, CFHR1, -2, -3, -5*) ist obligat. Ferner sollte, falls sicher durchführbar, eine Nierenbiopsie zur histologischen Diagnosesicherung angestrebt werden.

## Therapie

Eine erfolgversprechende Therapie ist von der Krankheitsursache abhängig [2]. Ist die TMA durch eine sekundäre Aktivierung des alternativen Wegs des Komplementsystems vermittelt, ist die Behandlung der Grunderkrankung essenziell. Eine suffiziente Einstellung des Blutdrucks hilft, eine sekundäre Endothelaktivierung von einer anderen Ursache zu unterscheiden. Eine antikörpermediierte TMA kann akut mit Plasmaaustausch behandelt werden, macht jedoch eine immunsuppressive Therapie notwendig. Gleiches gilt für eine erworbene TTP.

Eine Cobalamindefizienz soll mit einer Vitamin B_12_-, Folsäure- und Betainsubstitution behandelt werden. Eine medikamentös-toxisch ausgelöste TMA sollte nach Absetzen des suszipierten Medikaments reversibel sein. Das STEC-HUS wird supportiv behandelt, der Benefit eines Plasmaaustauschs ist fraglich. Eine durch Störungen des Gerinnungssystems ausgelöste TMA wird genauso wie die angeborene TTP mit Plasmainfusionen behandelt. Eine angeborene Störung der Regulation des Komplementsystems (aHUS/komplementvermittelte TMA) kann mit Plasmainfusionen oder -austausch sowie mit „anti-complement agents“ behandelt werden. Derzeit ist in Österreich nur der Antikörper Eculizumab gegen C5 Konvertase zugelassen. Substanzen, die in einem früheren Level der Komplementkaskade eingreifen oder auch den klassischen oder den Lektinweg blockieren, sind derzeit noch in klinischer Testung [15].

Eine medikamentös-toxisch ausgelöste TMA ist nach Absetzen des Medikaments reversibel

Zusammenfassend ist zu erwähnen, dass es bei den verschiedenen TMA rasch zu einem dramatischen Krankheitsverlauf kommen kann, weshalb die schnelle Erfassung der Erkrankung und der zugrunde liegenden Ursache sowie ein baldiger Transfer in eine spezialisiertes Zentrum für das Outcome des Patienten essenziell sind.

## Fazit für die Praxis

- TMA ist als mechanische Hämolyse, Thrombopenie und akutes Nierenversagen definiert.
- Ursächlich für das aHUS ist eine Dysregulation des Komplementsystems.
- Der Krankheit kann einen milden (unbemerkten) bis schweren Verlauf mit der Notwendigkeit einer intensivmedizinischen Intervention nehmen.
- Es gibt primäre und sekundäre TMA, wobei letztere wesentlich häufiger sind.
- Sekundäre TMA werden durch eine suffiziente Behandlung der Grundkrankheit behandelt.
- Bei primären TMA ist eine Substitution von komplementregulatorischen Proteinen durch Plasma oder ein Plasmaaustausch notwendig. In schweren Fällen ist eine Therapie mit „anti-complement agents“ notwendig.
